# Effect of Gum Arabic (*Acacia senegal*) on C-reactive protein level among sickle cell anemia patients

**DOI:** 10.1186/s13104-020-05016-2

**Published:** 2020-03-18

**Authors:** Lamis AbdelGadir Kaddam, Anas Suliman Kaddam

**Affiliations:** 1 Department of Physiology Faculty of Medicine, Alneelain University, P.O. Box: 11121, 12702 Khartoum, Sudan; 2 Department of Immunology, Institute of Tropical Medicine, Sudan Academy of Sciences, Khartoum, Sudan

**Keywords:** Gum Arabic, Sickle cell, Inflammation, CRP and butyrate

## Abstract

**Objectives:**

Inflammation is ongoing process among sickle cell anemia even during steady state. C reactive protein (CRP) is cardinal marker that utilized widely as inflammatory indicator. Gum Arabic (GA) is gummy exudates from *Acacia senegal* tree. Fermentation by colonic bacteria increases serum butyrate concentrations, so considered as prebiotic agent. Gum Arabic (GA) has anti-inflammatory activity through butyrate. Earlier we proved that regular intake of GA increased fetal hemoglobin and anti-oxidant capacity most likely through raised level of butyrate, which would ameliorate symptoms of sickle cell anemia. Best of our knowledge this is the first study conducted to investigate GA intake on inflammatory markers among sickle patients.

**Results:**

This was a retrospective study conducted on stored samples from trial of Gum Arabic and sickle cell anemia. Quantitative CRP was measured by Mindray BS 200 before and after Gum Arabic consumption for 12 weeks. Daily intake of GA significantly decreased C reactive protein level (P.V = 001) (95% CI 0.943–3.098). No correlation between CRP and age, fetal hemoglobin, hemolysis markers and white blood cells. Our findings revealed novel effect of GA as anti-inflammatory agent could be consumed as natural dietary supplement to modulate disease severity and downregulate inflammatory process.

Trial registration: ClinicalTrials.gov Identifier: NCT02467257. Registered 3rd June 2015

## Introduction

Sickle cell Anemia (SCA) is an autosomal recessive genetic disease that results from solitary point mutation in position 6 of the β-globin chain, leading to production of hemoglobin S (HbS) [1]. Africa is the main origin of the sickle (βS) mutations [2, 3]. Polymerization of deoxygenated sickle hemoglobin is the primary event in the molecular pathogenesis of sickle cell disease and is responsible for the vasoocclusive phenomena which is the hallmark of the disease [4]. Sickle cell disease (SCD) has long been recognized as an inflammatory condition and oxidative stress plays important role in pathophysiology of SCA [5]. SCA Patients have multiple indicators of an inflammatory response, including raised white cell counts, C-reactive protein (CRP) levels, cytokines, as well as activated monocytes, neutrophils, platelets, and endothelial cells [4]. Liver produced CRP as part of the acute phase reaction, in response to a host of pro inflammatory cytokines [6, 7]. CRP has wide acceptance as reliable indicator of systematic inflammation and tissue damage [7, 8]. Elevated levels of CRP, as a general marker of inflammation, have been previously reported in SCD patients and sickle mice [5, 9]. Elevated CRP in SCA patients may be in response to endothelium damage due to vascular endothelium blockage by sickle erythrocytes [5]. Further, it seem like CRP is elevated even during free crisis time i.e. among steady state condition [6, 10].CRP elevation during steady state may be attributed to sub clinical vaso occlusive episodes, which raise covert inflammatory response [10]. This response is mediated by cytokines primarily IL6 [10].

The US FDA recognized Gum Arabic (GA) as one of the safest dietary fibres [11, 12].GA is indigestible for both human and animals; its fermentation by colonic intestinal bacteria leads to formation of various degradation products, such as short-chain fatty acids [13]. Gum Arabic ingestion increases serum short chain fatty acid concentration, mainly butyrate and propionate [11, 14]. Serum butyrate concentration increased following administration of GA in healthy subjects [11, 15]. Butyrate has a potent anti-inflammatory effect. It decreases the pro-inflammatory cytokine expression through inhibition of NFκB [16, 17].Oral intake of GA has been shown to provide several health benefits [18], such as prebiotic effects [12]. GA has immune-modulatory [13, 19], anti-inflammatory [20], and antioxidant properties [11, 12, 21, 22].

We hypothesized GA degradation delivers short chain fatty acids, which in turn have been shown to stimulate fetal hemoglobin expression in RBCs as studied previously [23]. Also, serves as anti-inflammatory agent through Short Chain Fatty Acids (SCFA) production and provide some protection against damaging effects of inflammation and vaso-occlusive crisis. The present study tested whether Gum Arabic may influence the CRP level.

To the best of our knowledge, this is the first study conducted to investigate the effect of oral administration of GA on inflammatory markers in sickle cell anemia patients.

## Main text

### Methods

This is retrospective study conducted on stored blood samples of GA and Sickle cell clinical trial. Entry criteria, clinical monitoring, and laboratory measurements have been described in detail previously [23]. Patients were in steady state as define as crisis- free period for 3 weeks and 3 months or more after last blood transfusion [10]. Blood samples were collected before administering GA and after 12 weeks, as stated in the trial protocol [22].

Two ml in EDTA container, Three ml in plain container. The serum and plasma was separated by centrifugation and stored at − 85 °C. Blood samples were stored between twelve and eighteen months prior to analysis. Studies revealed that CRP can remains stable more than ten years when kept at/less than − 80 °C [24, 25].

### Gum Arabic administration

GA dose and administration were described in details in the previous report [23]. Properties and composition of GA are listed elsewhere [21].

Quantitative CRP was measured by Mindray BS 200 using turbidimetry method and expressed in mg/L. The principle of the test: Determination of the concentration of CRP through photometric measurement of immunocomplex between antibodies of CRP and CRP present in the sample, the absorbency increase is directly proportional to the concentration of CRP [26].

Data were analyzed using SPSS version 24. Paired samples T test was used to compare between pre and post intervention results. Person correlation was utilized to study correlation between contentious variables. P values equal or less than 0.05 was considered significant.

### Results

Thirty-four samples were available for CRP analysis before and after GA administration. Patients’ background characteristics are presented in Table 1. All were Sudanese; 50% were males (age 5 to 42 years). Five patients were on a stable dose of hydroxyurea 500 g per day. Duration of treatment was for 12 weeks.

**Table 1:** Demographics and baseline characteristics

Characteristics	Mean	SD	Median	Minimum	Maximum
Age	15.65	8.9	14	5	42
Gender	17(50%) Male				
Base line weight (Kg)	35·34	1.68	35	13	63
Base line height (Cm)	147·1	23**·**21	154**·**5	107	190
Hb (g/dL)	7.28	1.105	7	11	5.5
Hb F (%)	7.83	6.53	6.2	.00	29.60
Hb S (%)	88.89	6.22	91	68.00	97.00
CRP (mg/L)	4.22	5.89	2.3	0.10	27.80
WBCs (10^3^/uL)	14.51	4.45	13.8	7.60	26.00
LDH (U/L)	688.19	220.44	630	352.00	1250.00

**Fig. 1:**
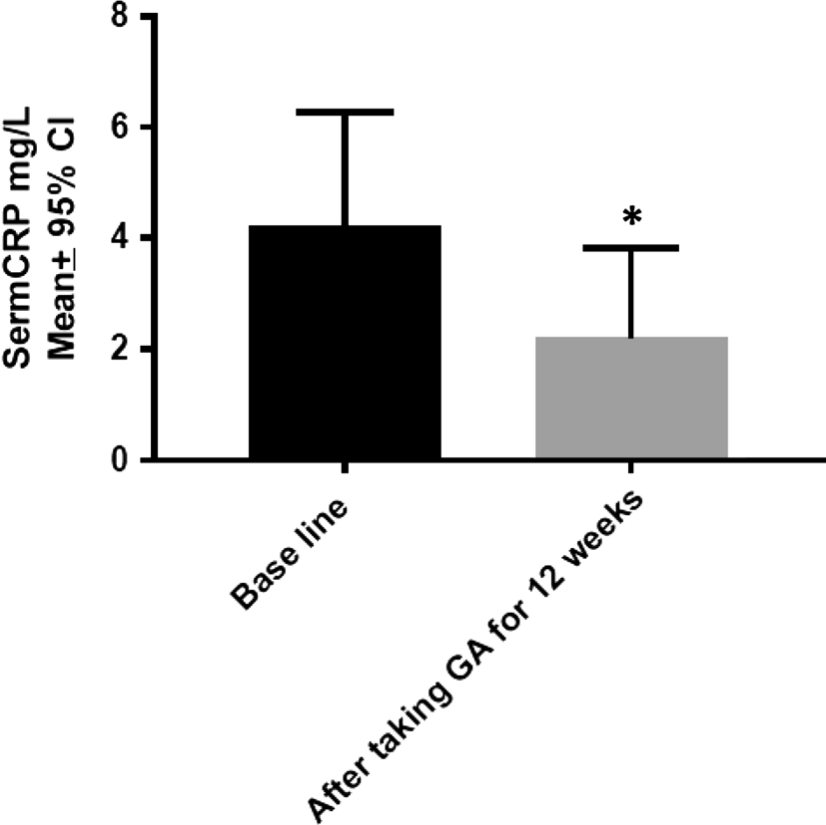
Effect of GA intake on CRP (P value = 0.001). *Indicates significant difference from baseline

Daily oral intake of GA significantly decreased CRP level (Fig. 1).

CRP showed no significant correlation with age, HbF level, TWBCs counts, Platelets count, and LDH level (Table 2).

**Table 2:** Correlation of different biomarkers with CRP level among SCA patients

Bio marker	Person correlation	P value
Age	− .091	0.608
Hb F	− .133	0.452
Hemoglobin	.169	0.339
WBCs	.155	0.383
Platelets	− .124	0.486
Reticulocytes	.146	0.589
LDH	− .300	0.101
Urea	− .180	0.333
Creatinine	.070	0.710

### Discussion

Sickle cell disease is the most common hemoglobin defect around the globe, with a high incidence in sub-Saharan Africa [27]. There is strong evidence generating a close connection between chronic inflammatory processes and sickle cell disease [28, 29]. Which it seems as inherent characteristic of sickle endothelia cells [29]. Inflammation has fundamental role in many comorbidity and mortality associated with SCD like acute chest syndrome for example. CRP is the most commonly assessed marker for acute and chronic inflammation [28]. In the current study, we revealed no significant relation between CRP level and Hb F (Table 2). Our results are comparable to earlier study, who interpret their results to other external factors like inflammation and vaso-occlusion due to SCA [6]. Monocytes, neutrophils, and platelets are also actively involved in the various adhesive interactions and clinical manifestations [30]. Chronic hemolysis plays major role in inflammation, among steady-state HbSS patients likely through subclinical vascular endothelial injury and transient vasoocclusive events [9]. Conversely, none of hemolytic markers (LDH, Reticulocyte counts, Hb concentration) correlated statistically with CRP level in this study (Table 2). CRP level could be activated by other pro-inflammatory cytokines such as Tumor Necrosis Factorα and IL-1b [9], which found consistently elevated among SCA patients [31]. These results were confirmed later by other investigators who found no significant difference between TNFα and other inflammatory cytokines level between SCA in steady state and in vaso-occlusive crisis [30].

GA significantly decreased CRP level (Fig. 1). This novel effect of GA may be of great importance, since inflammation is a cardinal component of the pathophysiology of SCD [32].

Earlier studies reveled GA anti-inflammatory effects as it decreased several inflammatory markers as TNFα, ESR and CRP [19, 20]. Reduction of CRP level could be accredited to GA prebiotics properties. Since several studies proposed that alteration in gut microbiota can alleviate inflammation [33–35]. SCFAs in particular butyrate have strong anti-inflammatory effect [16, 17, 36].

GA clinical trial among sickles induced HbF production [23] and this may have a role in reduction of ongoing inflammatory process and decreases CRP level. Nevertheless, we found no significant correlation between CRP and fetal hemoglobin (Table 2). On the other hand, there is strong relation concerning oxidative stress and inflammation and both are linked to SCD pathogenesis [5, 32, 37–39]. Earlier we documented GA exhibited strong anti-oxidant properties among SCA patients [22]. Therefore, reduction in CRP could be secondary to drop in oxidative stress markers. Numerous antioxidant therapies elicit anti-inflammatory responses [32].

In conclusion, our results reveled that inflammation among Sickle cell patients is ongoing process even during steady state period. GA significantly decreased CRP level, findings revealed an innovative effect of GA, which might be consumed as natural dietary fiber to attenuate inflammation in SCD patients and other pathogenesis linked with inflammatory process.

## Limitations

The short trial duration precludes us to confirm the clinical significance of our results in modulation of disease severity and related mortality. The study is single arm with no control group. The inference of our findings, that GA displays anti-inflammatory action among SCA patients. Longer and multi arm studies are recommended to validate our findings.

## Data Availability

The datasets used and/or analyzed during the current study are included in the main text. Further data can be obtained from the corresponding author on reasonable request.

## Abbreviations

CRP: C reactive protein
GA: Gum Arabic
Hb: Hemoglobin
HbF: Fetal hemoglobin
IL6: Interleukin 6
LDH: Lactate dehydrogenase
SCD: Sickle cell disease
SCA: Sickle cell anemia
